# Ligation of α-Dystroglycan on Podocytes Induces Intracellular Signaling: A New Mechanism for Podocyte Effacement?

**DOI:** 10.1371/journal.pone.0005979

**Published:** 2009-06-19

**Authors:** Nils P. J. Vogtländer, Henk Jan Visch, Marinka A. H. Bakker, Jo H. M. Berden, Johan van der Vlag

**Affiliations:** 1 Nephrology Research Laboratory, Nijmegen Centre for Molecular Life Sciences, Division of Nephrology, Radboud University Nijmegen Medical Centre, Nijmegen, The Netherlands; 2 Department of Biochemistry, Nijmegen Centre for Molecular Life Sciences, Radboud University Nijmegen Medical Centre, Nijmegen, The Netherlands; University of Birmingham, United Kingdom

## Abstract

**Background:**

α-Dystroglycan is a negatively charged glycoprotein that covers the apical and basolateral membrane of the podocyte. Its transmembrane binding to the cytoskeleton is regulated via tyrosine phosphorylation (pY892) of β-dystroglycan. At the basolateral side α-dystroglycan binds the glomerular basement membrane. At the apical membrane, it plays a role in the maintenance of the filtration slit. In this study, we evaluated whether ligation of α-dystroglycan with specific antibodies or natural ligands induces intracellular signaling, and whether there is an effect on podocyte architecture.

**Methodology/Principal Findings:**

Conditionally immortalized podocytes were exposed *in vitro* to antibodies to α-dystroglycan, and to fibronectin, biglycan, laminin and agrin. Intracellular calcium fluxes, phosphorylation of β-dystroglycan and podocyte architecture were studied. Antibodies to α-dystroglycan could specifically induce calcium signaling. Fibronectin also induced calcium signaling, and led to dephosphorylation of pY892 in β-dystroglycan. Ligation of α-dystroglycan resulted in an altered actin architecture, a decreased number of podocyte pedicles and a more flattened appearance of the podocyte.

**Conclusions/Significance:**

We conclude that ligation of α-dystroglycan on podocytes induces intracellular calcium signaling, which leads to an altered cytoskeleton architecture akin to the situation of foot process effacement. In particular the ability of fibronectin to induce intracellular signaling events is of interest, since the expression and excretion of this protein is upregulated in several proteinuric diseases. Therefore, fibronectin-induced signaling via dystroglycan may be a novel mechanism for foot process effacement in proteinuric diseases.

## Introduction

Podocytes are highly specialized glomerular visceral epithelial cells that cover the outside of the glomerular basement membrane [1]. Podocytes together with the glomerular basement membrane and the glomerular endothelium constitute the size and charge selective filter of the glomerulus. The podocyte cell body forms primary and secondary extensions, which form foot processes that interdigitate with those of neighboring podocytes and that are attached to each other by the slit diaphragm. In many glomerular diseases with proteinuria, podocyte foot processes are effaced, as their extensions are lost.

Dystroglycan was identified as a component of the dystrophin glycoprotein complex, which is involved in muscular dystrophies [2], [3]. Thereafter, dystroglycan was described as a component in epithelial cells [4], [5], and we have shown that dystroglycan is expressed by podocytes, both at the basolateral and the apical cell membrane [6], [7]. The dystroglycan encoding gene *DAG1* was first cloned and sequenced in 1992. Dystroglycan is post-translationally cleaved into the extracellular α subunit and the integral membrane β subunit, which non-covalently interact [2]. Dystroglycan provides a link between the extracellular matrix like the glomerular basement membrane to the actin cytoskeleton of the podocyte. α- Dystroglycan binds to laminin G modules that are present in laminin and agrin, whereas β-dystroglycan is connected to the actin cytoskeleton via dystrophin or its ubiquitous homologue utrophin in the kidney [6], [8], [9]. α-Dystroglycan undergoes massive O-mannosyl glycosilation with terminal sialic acids, which serve by virtue of their negative charge as an anti-adhesion layer on the apical podocyte membrane, analogous to podocalyxin, a sialic acid-rich glycoprotein that also covers the apical cell membrane of the podocyte [7]. The glycosilation of α-dystroglycan is necessary for its binding to laminin G modules [8]. We have shown previously that deglycosilation of α-dystroglycan by reactive oxygen species results in a loss of binding to agrin and laminin [10].

The small leucine-rich repeat at the C-terminal tail of α-dystroglycan has been shown to bind to chondroitin sulfate A and/or C on biglycan [11]. Biglycan is a small leucine-rich proteoglycan with chondroitin sulfate or dermatan sulfate side chains. It has been shown to be predominantly localized in the tubular interstitium and intrarenal arteries. Minimal expression of biglycan in the mesangial matrix has been described for normal human kidney and various glomerular diseases [12]–[16]. TGF-β-mediated mesangial upregulation of biglycan has been described in Thy-1 mediated membranoproliferative glomerulonephritis in the rat [17]. TGF-β induces fibrosis and as a consequence it is especially co-localized with collagen type I in fibrosis [13].The assembly of chondroitin sulfate side chains decreases after exposure to puromycin-aminonucleoside and dexamethasone [18], [19]. Biglycan is not detectable in the urine of patients with glomerular diseases [15].

It was recently shown that the C-terminal domain of muscle α-dystroglycan can also bind to fibronectin and fibrinogen [20]. Fibronectin is a 500 kDa protein that consists of two similar 250 kDa subunits linked via a disulfide bond. By post-translational processing plasma and tissue isoforms are formed. An increased expression of both plasma and tissue fibronectin has been documented in various glomerular diseases like diabetic nephropathy, puromycin-aminonucleoside and adriamycin nephrosis [21]–[24]. Furthermore, urinary excretion of fibronectin has been shown to correlate with proteinuria in IgA-nephropathy, membranous glomerulopathy, and diabetic nephropathy and with glomerulosclerosis in lupus nephritis and chronic glomerulonephritis [25]–[27].

The extracellular N-terminal domain of β-dystroglycan interacts with α-dystroglycan, whereas the intracellular C-terminal domain interacts with utrophin [28]–[30]. In particular, a PPxY motif including a tyrosine residue at position 892 in β-dystroglycan in the C-terminal domain is necessary for interaction with WW domains in utrophin [28]–[30]. WW domains are 30–40 amino acid motifs containing two conserved tryptophan residues, that bind to proline-rich sequences [31]. The interaction of β-dystroglycan with dystrophin/utrophin is stabilized by a helix-turn-helix motif, i.e. EF hand, close to the PPxY motif in dystrophin/utrophin [29], [32]. Furthermore, dephosphorylation of Y892 (pY892) in β-dystroglycan enables binding to utrophin/dystrophin [30], [33]. Phosphorylation of β-dystroglycan enhances recruitment of SH2/SH3 domain containing proteins like c-Src, Fyn, Csk, NCK, SHC and Grb-2, which all are involved in signaling events [34], [35]. This suggests a role for dystroglycan in signaling.

In this study, we investigated whether ligation of α-dystroglycan by monoclonal antibodies against α-dystroglycan and natural ligands for α-dystroglycan, such as laminin, agrin, biglycan and fibronectin, leads to intracellular signaling as assessed by measuring calcium fluxes and to changes in the phosphorylation status of β-dystroglycan at position 892 (Y892). In addition, we investigated whether α-dystroglycan ligation had an impact on actin cytoskeleton architecture and podocyte morphology.

## Results

**Figure 1:**
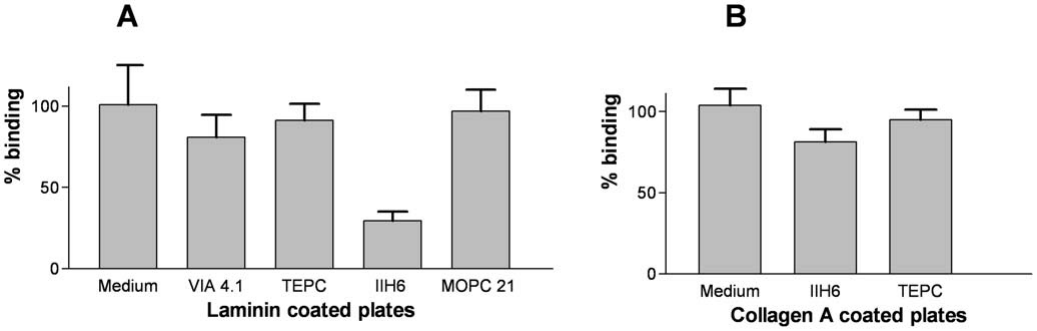
Inhibition of podocyte binding to laminin by anti α-dystroglycan monoclonal antibodies IIH6 and VIA 4.1. Podocytes were seeded on laminin (A) or collagen A coatings (B) after pre-incubation with either monoclonal antibody IIH6 using TEPC as an isotype control, or with VIA 4.1 using MOPC 21 as an isotype control. Monoclonal antibody IIH6 specifically inhibits podocyte binding to laminin, but not to collagen A.

The binding of cultured mouse podocytes to EHS laminin, agrin, collagen A and Matrigel coated plates was quantified with the hexosaminidase assay. Podocytes were pre-incubated with α-dystroglycan specific antibodies, IIH6 or VIA4.1, directed to carbohydrate epitopes on α-dystroglycan, prior to seeding on the coatings. It is known that monoclonal antibody IIH6 inhibits the binding of α-dystroglycan to laminin G modules, whereas monoclonal antibody VIA 4.1 does not [4], [36]. Indeed, the number of podocytes binding to laminin decreased after pre-incubation with IIH6, but remained unaltered after pre-incubation with VIA4.1 (Figure 1A). Pre-incubation with isotype controls for either of the α-dystroglycan antibodies (TEPC and MOPC respectively) did not affect the binding. As expected, similar results were obtained with agrin as coated ligand (data not shown). However, the binding of podocytes to collagen A, which is comprised of type I collagen that does not contain laminin G modules, was unaltered by the addition of either of the two α-dystroglycan specific antibodies (Figure 1B). Furthermore, the binding of podocytes to Matrigel© which is considered as a surrogate basement membrane preparation containing a mixture of several extracellular matrix molecules, could not be prevented by MoAb IIH6 (data not shown). Therefore, monoclonal antibody IIH6 specifically inhibits the binding of mouse podocytes to laminin EHS and agrin, indicating an important role for α-dystroglycan for the binding of podocytes to laminin G modules containing proteins such as laminin and agrin in the glomerular basement membrane.

**Figure 2:**
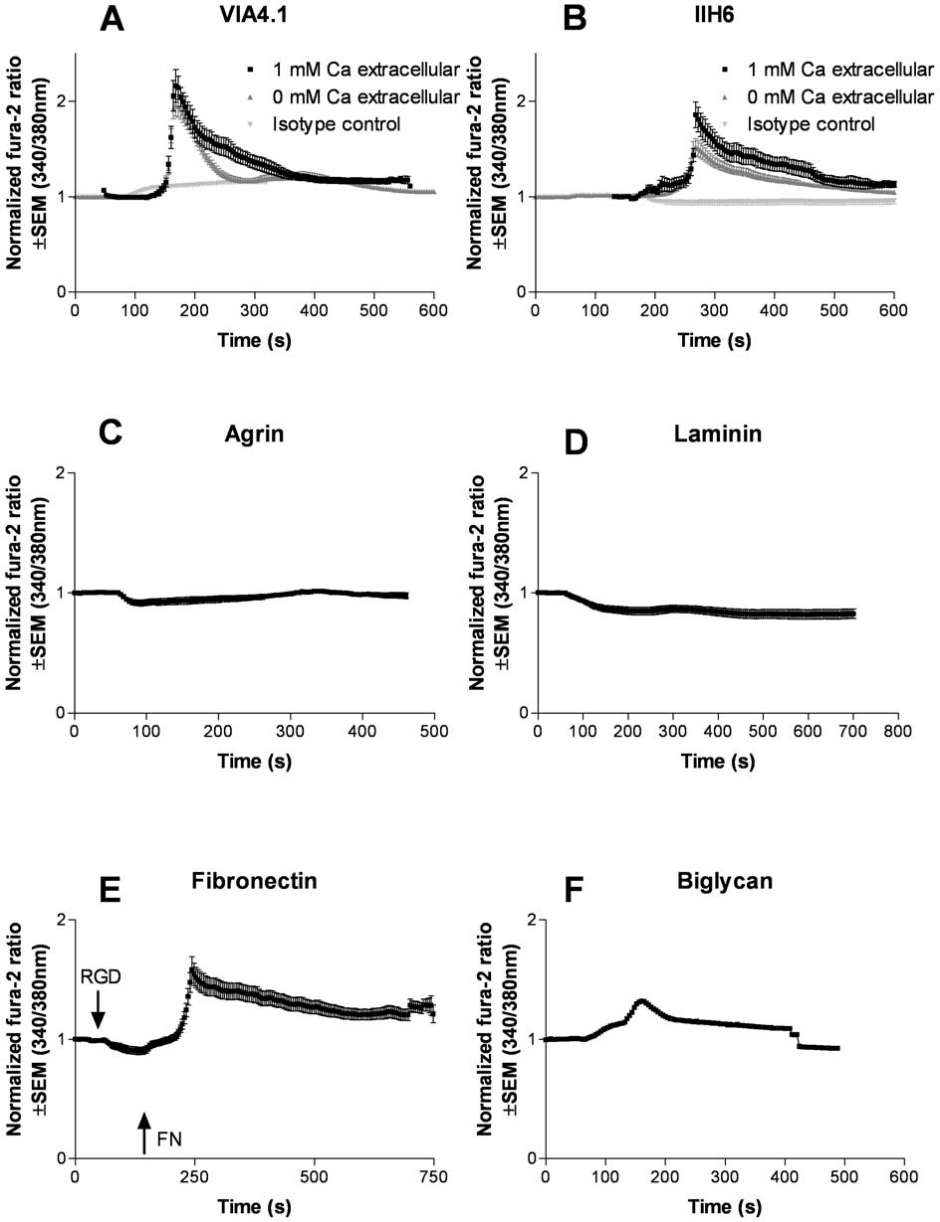
Calcium fluxes in cultured mouse podocytes after ligation of α-dystroglycan. Mouse podocytes were differentiated on collagen A coatings for 2 weeks. Intracellular calcium concentrations were monitored by the fura-2 method, with and without extracellular calcium. Both α-dystroglycan specific monoclonal antibodies induced calcium fluxes, most likely from intracellular stores. Isotype controls were negative (A, B). The laminin G modules-containing proteins agrin and laminin were unable to induce a calcium influx in podocytes (C, D). From the natural ligands of α-dystroglycan tested, fibronectin induced the most pronounced calcium influx. Note that blocking of potential integrin binding sites with a RGD peptide did not influence the fibronectin effect (E). The proteoglycan biglycan induced a modest calcium influx (F).

**Figure 3:**
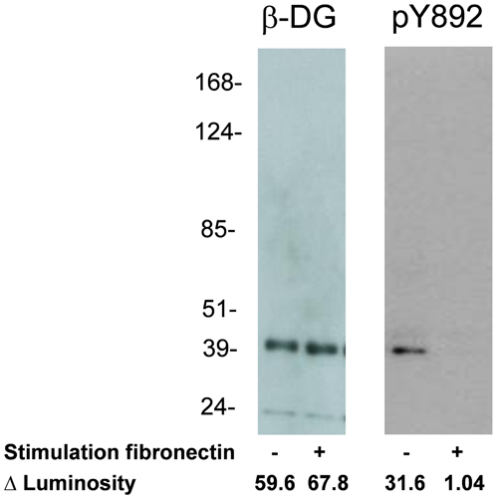
Fibronectin stimulation of cultured mouse podocytes leads to a decrease of pY892 on β-dystroglycan. Cultured mouse podocytes were incubated for 3 minutes with fibronectin after which a decrease of pY892 was found in Western blot analysis of whole podocyte cell lysates (right panel), whereas the total amount of β-dystroglycan was not affected (left panel). Total β-dystroglycan was probed with NCL-BDG and pY892 was probed with anti-pY892.

**Figure 4:**
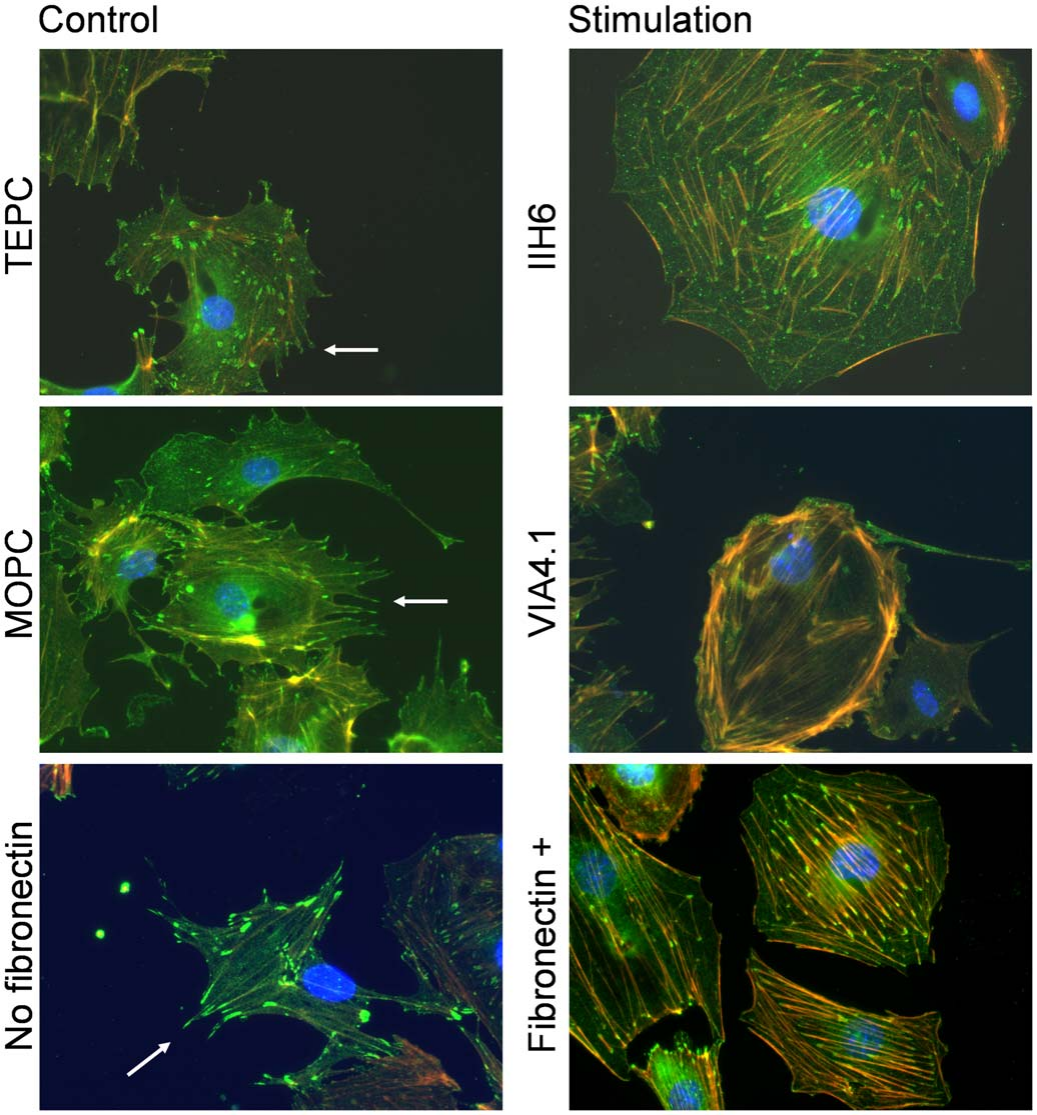
Ligation of α-dystroglycan results in a loss of pedicles in cultured mouse podocytes. Differentiated podocytes on collagen coatings were incubated with either monoclonal antibodies IIH6 or VIA 4.1, their respective isotype controls (TEPC or MOPC) or fibronectin. The podocyte pedicles (arrow) are probed with antibodies against Mena (green, which also localizes along stress fibers and in focal contacts at the tips of stress fibers [58], [65]). The pedicles were lost after 6 hours of incubation with the antibodies. As these pedicles may be the *in vitro* manifestation of foot processes, their retraction may imply foot process effacement. Percentage of podocytes with smooth surfaces were counted, which yielded for IIH6 60% *versus* TEPC 30% (χ^2^ = 20.8, p<0.001), for VIA4.1 51% *versus* MOPC 28% (χ^2^ = 9.01, p<0.01) and fibronectin 38% versus control 30% (χ^2^ = 1.3, p = 0.25). (Red = phalloidin (actin); blue = DAPI (DNA)).

Next, we analyzed whether α-dystroglycan on mouse podocytes could mediate outside-in signaling. The ability of monoclonal antibodies IIH6 and VIA 4.1 and their respective isotype controls to induce calcium fluxes was assessed by the fura 2 method, which measures the cytosolic calcium concentration. Both monoclonal antibodies could specifically induce calcium signaling after ligation of α-dystroglycan, whereas the isotype controls did not (Figure 2A, B). The peak of the cytosolic calcium concentration was similar in the presence or absence of extracellular calcium, indicating that the cytosolic calcium influx was derived from the intracellular calcium stores. Although these experiments indicate that ligation of α-dystroglycan mobilizes intracellular calcium stores, this induction by specific antibodies is however, not a physiological event. Therefore, the ability of natural ligands of α-dystroglycan to induce a similar rise in cytosolic calcium was investigated. Both laminin and agrin, which both can bind to glomerular α-dystroglycan [10], were not able to induce a rise in cytosolic calcium (Figure 2C, D). Biglycan induced a modest calcium influx (Figure 2F). Since the interaction of biglycan with muscle α- dystroglycan depends on the chondroitin sulfate side chains of biglycan [11], we also tested the effect of adding chondroitin sulfate A, B and C, and heparan sulfate. However, both chondroitin sulfate preparations as well as heparan sulfate did not induce calcium mobilization in the cytosol (data not shown). Since fibronectin is a known ligand of muscle α-dystroglycan, and a protein which is involved in several glomerular diseases [21]–[27], we evaluated the effect of fibronectin on calcium fluxes. Since we could not rule out that RGD motives in fibronectin could induce signaling via α_3_β_1_integrin, these RGD fibronectin receptor recognition sequences in integrins were blocked by pre-incubation with RGD peptide. We included RGD blocking, although α3β1integrins are mainly expressed at the basal and not at the apical side of podocytes. Addition of RGD peptide did not result in calcium fluxes. Fibronectin, after blocking of α_3_β_1_integrin with RGD peptides, induced the strongest cytosolic calcium influx of all 4 endogenous ligands of α-dystroglycan tested (Figure 2E). This finding indicates that fibronectin can induce calcium fluxes in podocytes, via other molecules than integrins. To further investigate whether the dystroglycan complex is involved in fibronectin-induced calcium fluxes in podocytes, its effect on the phosphorylation state of Y892 in β-dystroglycan was investigated. It appeared that stimulation of podocytes by fibronectin (concomitantly with blocking with RGD peptide) led to a decrease in phosphorylation of Y892, while the total amount of β-dystroglycan was unaltered (Figure 3). This latter finding suggests that α-dystroglycan is involved in fibronectin-induced calcium influxes in podocytes. We have also studied the expression level of pY892 in renal sections of control rats and rats with adriamycin nephropathy, with normal and effaced podocytes, respectively. We applied several antigen retrieval techniques, and an epitope preservation approach by inclusion of phosphatase inhibitors. However, despite an additional signal amplification, the detectable expression levels of pY892 in both control and adriamycin rats were extremely low and, therefore, not conclusive (data not shown), which seems in contrast to our clear findings with Western blot analysis (Figure 3). However, a similar discrepancy between immunofluorescence and Western blot analysis has been observed on skeletal muscle by others [37]. It has been described that differentiation of conditionally immortalized podocytes induces process (filopodia) formation [38]. In figure 4 we show the effect of the anti-α-dystroglycan monoclonal antibodies and fibronectin on the morphology of podocytes and actin cytoskeleton. All ligands induced a retraction of filopodia and a reorganization of the cytoskeleton. Statistical analysis of these effects revealed a significant effect for the anti-α-dystroglycan monoclonals IIH6 and VIA 4.1, but only a trend for fibronectin (Table 1). However, in several fibronectin-stimulated cells retraction of filopodia was clearly visible as shown in the lower panels of figure 4. This reorganization of the actin cytoskeleton may be a situation reminiscent of foot process effacement. These data suggest that fibronectin-induced signaling events, mediated by the dystroglycan complex leads to an altered podocyte architecture.

**Table 1:** Percentages of podocytes with smooth, intermediate or filopodia rich surfaces after *in vitro* ligation with α-dystroglycan specific antibodies, fibronectin and their respective controls.

	IIH6**	TEPC 183**	VIA4.1***	MOPC21***	FN+	FN-
Filopodia rich*	12	25	17	28	18	23
Intermediate*	28	45	32	44	44	46
Smooth*	60	30	51	28	38	30

* Smooth 0–0.2 filopodia/5 µm, intermediate 0.2–1 filopodia/5 µm or filopodia rich ≥1 filopodia/5 µm cell border.

** Significantly different when comparing frequencies in both columns; IIH6 vs. TEPC183 χ^2^ = 20.8, p<0.001.

*** VIA4.1 vs. MOPC21 χ^2^ = 9.01, p<0.01 Fibronectin+(FN+) vs. control (FN-) χ^2^ = 1.3, p = 0.25.

## Discussion

α-Dystroglycan is a negatively charged glycoprotein, which covers the apical and basolateral membrane of the podocyte. At the basolateral side α-dystroglycan binds laminin G modules in agrin and laminin. The α_3_β_1_integrin is also expressed at the basolateral side of podocytes, where it extracellulary binds to laminin, fibronectin and collagen. In this study we investigated the importance of α-dystroglycan for the binding of podocytes to laminin, agrin and several other extracellular matrix molecules with a new binding assay. Binding of monoclonal antibody IIH6 but not monoclonal antibody VIA4.1 inhibited the binding of podocytes to laminin and agrin. Our findings with podocytes are similar to the data for the binding of Schwannoma cells to laminin as previously described [36]. This is also underlined by the observation that monoclonal antibody VIA 4.1 did not inhibit the binding/growth of podocytes to collagen nor to laminin and agrin, since the carbohydrates recognized by VIA4.1 are not involved in the binding to laminin G moieties [4], [36]. However, no impairment of binding of podocytes to collagen type I coated plates was observed after incubation with monoclonal antibody IIH6. Furthermore, binding of podocytes to Matrigel-coated plates also could not be inhibited by IIH6, which not unexpectedly suggests that podocytes also can bind to additional molecules without laminin G modules. As over 50% of the binding of podocytes to laminin was lost by the addition of monoclonal antibody IIH6, it can be concluded that for binding to laminin α-dystroglycan on podocytes is of equal or even greater importance than α_3_β_1_integrins. Therefore, our *in vitro* findings suggest that α-dystroglycan represents an important mechanism for podocytes to anchor to the glomerular basement membrane.

At the apical domain of the podocyte the assumed function of the dystroglycan complex is maintenance of the filtration slit by its negative charge [7]. In this study, we investigated whether dystroglycan plays a role in outside-inside signaling events. Indeed, we observed a specific cytosolic calcium influx in cultured mouse podocytes after ligation of α-dystroglycan by two different anti α-dystroglycan antibodies. This rise in the cytosolic calcium concentration comes mainly from the intracellular calcium stores, since it was not dependent on the extracellular calcium concentration. This increase in cytosolic calcium is presumably mediated via inositol-1,4,5 tri-phosphate in the endoplasmatic reticulum [39].

Since induction of calcium fluxes by these anti α-dystroglycan specific antibodies is not a physiological event, we were interested in the effects of natural ligands of dystroglycan like, laminin, agrin, biglycan and fibronectin. From these ligands fibronectin induced the most pronounced signal, biglycan induced only a weak increase in cytosolic calcium, while agrin and laminin were not able to induce such an effect. This difference may be related to the fact that the function of α-dystroglycan on the basal side of the podocyte is different from the function at the apical side. Furthermore, clustering of α-dystroglycan can be induced by the antibodies, fibronectin and biglycan, as they may all serve as bivalent ligands [20]. In contrast, agrin and laminin probably serve as monovalent ligands. Tandem LG modules are necessary for α-dystroglycan binding. Since agrin contains only 3 LG modules, only one tandem is available [40]. Laminin possesses 5 LG modules which enable the formation of maximally two LG tandems. However, their spatial relationship within the molecule may be to close to cross link α-dystroglycan [41], for review see [42]. The fibronectin induced rise in cytosolic calcium can in theory be mediated via many complexes. The integrin α_3_β_1_ can bind fibronectin via a RGD module, and is intracellulary linked to integrin linked kinase (ILK) which is involved in regulation of the actin cytoskeleton, morphology and cell survival via signaling events [43], [44]. However, the involvement of integrins in the fibronectin-induced rise in cytosolic calcium was excluded by blocking with an RGD peptide. Furthermore, probing of podocytes with fibronectin induced a dephosphorylation of β-dystroglycan, which makes a role for α-dystroglycan in fibronectin induced signaling likely. Immunoprecipitation studies in our laboratory have indeed showed that glomerular α-dystroglycan can bind fibronectin (data not shown), confirming previously published observations [20]. Intact fibronectin chains (∼250 kDa) can only pass the glomerular basement membrane if proteinuria is present, and indeed urinary fibronectin correlates with proteinuria [25]–[27]. Importantly, tissue fibronectin is upregulated during several glomerular diseases [21]–[24].

The unraveling of the downstream signaling pathways after dystroglycan ligation in podocytes remains challenging. The PPxY domain of β-dystroglycan can recruit SH2/SH3 domain-containing proteins like c-Src, Fyn, Csk, NCK, SHC and Grb-2. These interactions are enhanced by phosphorylation of pY892 [34]. From these, c-Src has been shown to be able to phosphorylate Y892 in c-Src overexpressing COS7 cells [37]. Also the expression of pY892 β-dystroglycan increases in COS7 cells when cultured on coatings of laminin, agrin or fibronectin [37]. Caveolin 1 and 3 co-express with β-dystroglycan in lipid raft domains, and bind when Y892 is phosphorylated. Caveolin 3 has been shown to inhibit the c-Src induced phosphorylation of Y892. [45]. Furthermore, the proline-rich SH3 region of Grb-2 binds at the C terminal side of β-dystroglycan [35]. p125^FAK^ has been shown to be linked to Grb-2, but not directly to β-dystroglycan [46]. Taken together, the dynamic role of β-dystroglycan in signaling seems to provide docking sites for multiple kinases when phosphorylated, or to provide a stable anchorage to the actin cytoskeleton when dephosphorylated. A similar phenomenon was illustrated by a study in which the Src family kinases were shown to be important for the stability of dystroglycan containing clusters induced by agrin [47]. β-dystroglycan also is able to bind ezrin, which also affects its interaction with the actin cytoskeleton [48]. Dystroglycan suppresses laminin 1 and -10/11 induced ERK activation via α_6_β_1_ integrin and direct linking of β-dystroglycan to the ERK-MAP kinase pathway has also been described in COS 7 cells [49], [50]. Recently, genetic interaction screens on presumed modifier genes for dystroglycan and dystrophin in many drosophila melanogaster mutants, added even more putative signaling pathways, like the Notch, TGF-β, EGFR, Semaphorin-Plexin, Frazzled-Netrin and Slit-Robo pathways [51].

Unfortunately, in co-immunoprecipitation experiments we could not find any binding of β-dystroglycan to Src, Grb2, p125^FAK^ and ezrin in rat kidney lysates (data not shown). So the signaling pathways linked to β-dystroglycan in podocytes remains subject for further investigation.

Alterations in podocyte shape and function are calcium dependent. This has been shown in the calcium switch model in podocytes, in which 90 minutes removal of extracellular calcium results in an increased paracellular flux of dextrans [52]. Mutations in the canonical transient receptor potential 6 (TRPC6) result in an increased amplitude of the calcium current from the extracellular calcium stores, and eventually leads to a form of autosomal dominant focal segmental glomerulosclerosis [53], [54]. However, the calcium flux induced via α-dystroglycan is derived from the intracellular calcium stores, which suggests that TRPC6 is not involved in these events. Furthermore, podocyte shape alterations can be induced by sialidase or puromycin aminonucleoside in rats; dephosphorylation of ezrin linked to podocalyxin via NHERF2 results in dissociation from the actin cytoskeleton and concomitant foot process effacement [55]. The involvement of β-dystroglycan in fibronectin-induced signaling is shown by the decrease in phosphorylation of Y892, which however enhances binding of the dystroglycan complex to the actin cytoskeleton via utrophin. The impact of this enhanced binding to actin was visualized by changes in podocyte morphology. We observed a reorganization of the actin cytoskeleton after ligation of α-dystroglycan, with monoclonal antibodies to α-dystroglycan and in a subset of podocytes with fibronectin. This was accompanied by a retraction of branches of cultured mouse podocytes. Similar observations have been made by Rüdiger et al. after exposure of mouse podocytes to protamin sulfate, which resulted in instant calcium influxes, and podocyte foot process retraction after 2 hrs [56]. Protamin sulfate inhibits the binding of α-dystroglycan to laminin and can also bind α-dystroglycan, *in vivo* injection results in immediate foot process effacement and endocytosis of α-dystroglycan [57]. Thus, the *in vitro* retraction of foot processes may reflect *in vivo* podocyte foot process effacement.

In summary, the binding of fibronectin to apical free accessible α-dystroglycan leads to signaling events in the podocytes, as indicated by a rise in cytosolic calcium and a dephosphorylation of β-dystroglycan, enabling utrophin and the actin cytoskeleton to bind and retract the podocyte foot process. This may represent a novel mechanism of podocyte foot process effacement in proteinuric disease.

## Methods

### Cell culture

Conditionally immortalized mouse podocytes (MPC-5, generously provided by Peter Mundel, Division of Nephrology, Albert Einstein College of Medicine, Bronx, NY, USA) were cultured essentially as described before [7], [38]. MPC-5 express α-dystroglycan at their apical and basal cell membrane [7]. Passages 12–17 were used in these experiments. Indirect immunofluorescence was performed as described previously [7].

Anti-Mena antibody (Becton Dickinson, Transduction Laboratories, San Jose, CA, USA) dilution 1∶40 and goat anti-mouse IgA FITC (Southern Biotech, Birmingham, Alabama, USA) dilution 1∶50 were used to probe the filopodia. [58]. Phalloidin-TRITC (Molecular Probes, Leiden, The Netherlands) dilution 1∶150, and DAPI (Molecular Probes) dilution 3*10^−5^ was used to visualize the actin cytoskeleton and DNA, respectively. Sections were investigated by a Leica DM 4000 B microscope (Leica Microsystems, Rijswijk, the Netherlands). Filopodia count on the cell border was studied as follows: The number of filopodia of 2 µm or longer was counted per 5 µm cell border. Cells were divided into 3 categories: smooth 0–0.2 filopodia/5 µm, intermediate 0.2–1 filopodia/5 µm or filopodia rich ≥1 filopodia/5 µm cell border. The frequencies in the groups were compared using χ^2^.

For the investigation of binding, propagated podocytes were seeded overnight on laminin EHS (Campro Scientific; Veenendaal, The Netherlands) or collagen A (Biochrom, Berlin, Germany) coated 96 microwell plates (Costar, Corning Life Sciences, Corning, NY) using 3000 podocytes/well with the addition of 1 µM α-dystroglycan specific monoclonal antibodies, IIH6 or VIA4.1 [59], or their respective isotype controls, MOPC 21 or TEPC 183 (Sigma, St Louis, MO). Notably, the glycoepitopes on glomerular α-dystroglycan are recognized by the antibodies IIH6 and VIA4.1 and expressed by cultured mouse podocytes [7], [60]. Podocytes were inversely centrifuged at 200 g for 1 minute and carefully rinsed three times with phosphate buffered saline (PBS, Merck, Darmstadt, Germany).

To evaluate the number of podocytes a hexosaminidase essay was performed in which the amount of the house keeping enzyme hexosaminidase is quantified by measuring the digestion of p-nitrophenyl-*N-*acetyl-β-D-glucosamidine [61]. In short: podocytes were incubated overnight at 37°C with 100 µl 50 mM citrate (Merck), 3.75 mM p-nitrophenyl-*N-*acetyl-β-D-glucosamidine (Sigma) and 0.25% Triton ×100 (Sigma). Thereafter, the reaction was blocked with a solution containing 50 mM glycine (Sigma) and 5 mM EDTA, pH 10.4, 150 µl/well. Optical density was measured at 405 nm. Two-tailed Student-*t* test was performed and *P*≤0.05 was considered significant.

### Measurement of calcium fluxes

Propagated podocytes were seeded on collagen A coated glass cover slips and differentiated for 3 weeks. Subsequently, cells were loaded with 5 mM fura-2-AM (Molecular Probes) and 0.025% pluronic-F127 (Molecular Probes), added to the medium of differentiated podocytes for 20 minutes at 37°C. Subsequently, they were washed and mounted in a bath-chamber in 137 mM NaCl, 3.6 mM KCl, 1 mM CaCl_2_, 1 mM MgCl_2_, 5,5 mM D-glucose and 10 mM Hepes-Tris buffer pH 7.4 (Sigma) at 37°C and placed under an inverted microscope (Nikon) attached to a videorate confocal microscope (Noran Instruments, Middleton, WI). Calcium-free measurements were performed in the same buffer without calcium and with 2 mM ethylene glycol tetra-acetic acid (EGTA, Sigma) added. The podocytes were excited 15 times/min at 340 and 380 nm, with an exposure time of 100 ms and single cell emission at 492 nm was monitored as a measure of the cytosolic free calcium concentration. Dynamic video imaging was carried out using the MagiCal hardware and Tardis software [62]. The effects of the addition of 1 µM biglycan (Sigma), 1 µM laminin, 1 µM IIH6, 1 µM VIA4.1, 1 µM MOPC 21, 1 µM TEPC 183 (Sigma), 1 µM heparan sulfate (Seikagaku), a mixture of 2 mg/ml chondroitin sulfate A, 1 µM B and C (Sigma), or 0.025 mg/ml rat fibronectin (250 kDa chains, Sigma) on calcium fluxes were evaluated. Only for the measurement of the effect of fibronectin on calcium fluxes, podocytes were prior to the addition of fibronectin maintained in serum-free medium for 24 hrs. Possible fibronectin binding sites on integrins were blocked with GRGDS peptide (160 µM, Sigma).

### Western blot analysis

Western blot analysis was performed according to the methods of Laemmli and as described recently [10], [63]. Whole podocyte cell lysates were blotted onto nitrocellulose and blocked with 5% non-fat dry milk (Biorad, Veenendaal, the Netherlands) in PBS. Phosphatase inhibitors, 10 mM NaF (Sigma) and 1 mM sodium orthovanadate (Sigma), were added to the buffers used during the whole procedure to prevent dephosphorylation [64]. Anti-pY892 (1∶50 BD Biosciences, San Jose, USA) and NCL-BDG (1∶10 Novocastra, Newcastle upon Tyne, United Kingdom) were used as primary antibodies. Peroxidase labeled goat anti mouse IgG_H+L_1∶5000, was used as secondary antibody.
